# Does Nutrition Knowledge Help? Heterogeneity Analysis of Consumers’ Willingness to Pay for Pre-Packed Mooncakes Labeled with the Smart Choice Logo

**DOI:** 10.3390/foods13244027

**Published:** 2024-12-13

**Authors:** Zeying Huang

**Affiliations:** Institute of Food and Nutrition Development, Ministry of Agriculture and Rural Affairs, Beijing 100081, China; huangzeying@caas.cn; Tel.: +86-13716627967

**Keywords:** Smart Choice logo, willingness to pay, pre-packed mooncakes, front-of-package nutrition labeling, premium

## Abstract

The Smart Choice logo (SCL), as an encouraging form of front-of-package nutrition labeling (FOPNL), helps consumers to choose low-oil, -salt, and -sugar mooncakes during the Mid-Autumn Festival. It is widely acknowledged that nutrition knowledge contributes to nutrition label use, but there has been little research on whether it helps enhance consumers’ willingness to pay (WTP). Our study aims to fill this gap by investigating 630 randomly selected Chinese adults from Jilin, Inner Mongolia, Shaanxi, Shandong, Henan, Sichuan, and Guangdong. The semi-double-bounded dichotomous choice contingent value method was selected to measure their WTP for pre-packed mooncakes with the SCL at 20 different premium levels, ranging from 0% to 95% of the price per unit. It was found that the respondents’ WTP decreased by 0.7% as the premium level increased by 1%, and the WTP of people from South China, those who were obese, and those with a high income was not sensitive to changes in premium. Nutrition knowledge played a negative moderating role, and the probability of the premium levels affecting WTP decreased by 1.0% for each 1 point increase in the nutrition knowledge level. These findings highlight the potential implications associated with SCL promotion and differentiated mooncake pricing, as well as the supply of healthier Chinese holiday foods.

## 1. Introduction

Diet-related chronic diseases not only threaten human health but also impose a substantial social burden. According to In Brief to The State of Food Security and Nutrition in the World 2024, global dietary risk factors such as a low intake of whole grains, fruits, and vegetables; an excessive intake of sodium; and a high intake of red and processed meat result in a high incidence of hypertension, diabetes, and hyperparathyroidism, for which the hidden treatment and care costs, as well as the lost productivity due to illness, are significant [1]. For developing healthy eating habits among residents, the World Health Organization (WHO) has advocated for the use of front-of-package nutrition labeling (FOPNL) on pre-packed foods to help consumers to easily choose healthier foods [2,3]. The FOPNL is simplified nutritional information presented on the front of food packages that provides information about the overall nutritional status or key nutrient content of food through symbols, graphics, or text, helping consumers make informed food choices [4]. It has recently been recommended by the WHO and the Codex Alimentarius Commission (CAC) [4,5].

**Figure 1:**
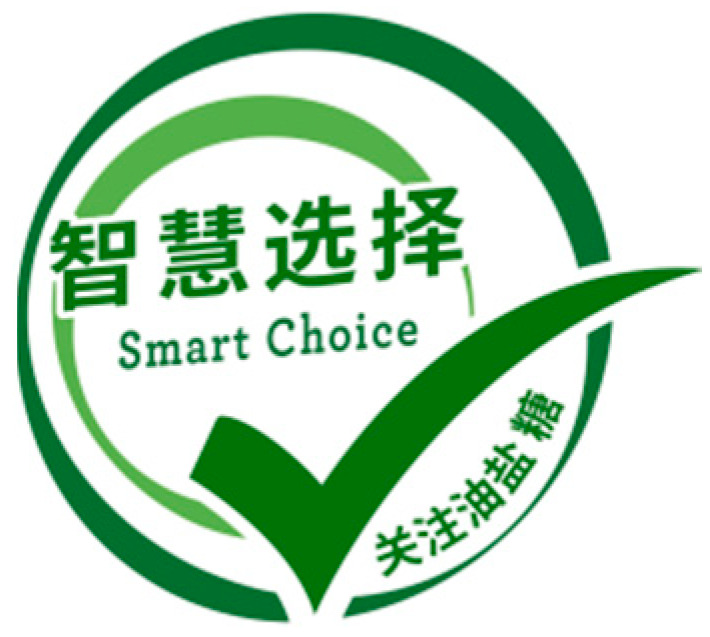
Smart Choice logo. Source: The Smart Choice logo standard for prepackaged food (T/CNSS001-2018). Note: The Chinese characters in the middle are Smart Choice; the Chinese characters in the lower right corner are attention to oil, salt, and sugar.

It was not until 2019 that China implemented the first interpretive FOPNL, the Smart Choice logo (SCL) (see Figure 1), while more than 40 countries, including Sweden, the United States, the United Kingdom, Singapore, the Netherlands, Chile, Australia, France, and Italy, have implemented their own FOPNL. French Nutri-Score labeling and Chilean warning labeling are examples of internationally representative FOPNL. The former is a rating-based FOPNL, with ratings of A~E and five colors representing the foods’ overall nutritional health [6,7]; the latter is a restrictive FOPNL comprising four text-based labels, such as high energy, high fat, high sugar, and high sodium labels, to alert consumers that the foods are unhealthy [8,9]. In contrast, the SCL, as encouraging FOPNL, was developed by the Chinese Nutrition Society to certify pre-packed foods that are low in salt, oil, and sugar by labeling them with a green check mark. The Smart Choice logo standard for prepackaged food (T/CNSS001-2018), a nutrition labeling group standard, divides all pre-packed foods into ten categories (cereal products, bean products, milk and dairy products, nuts and seeds, meat and similar products, aquatic products, egg products, vegetable and fruit products, beverages, and puffed snacks) [10]. The maximum content thresholds for fat, saturated fatty acids, total sugars, sodium, and added sugars in these foods are designated based on the lower quartile of the above nutrient content ranked from highest to lowest, which means that about 25% of food items on sale meet the SCL standard. T/CNSS001-2018 also stipulates that the SCL may be voluntarily displayed on the front packaging of food products that meet the standard. However, foods with the SCL are scarce in China, as the government does not mandate the use of the SCL, and it is at the discretion of healthy food manufacturers whether they adopt the logo.

Mooncakes are cakes containing cereal flour, oil, sugar, and fillings (e.g., lotus seed paste, salted egg, fruit, melon kernels, or pork), typically with a high content of oil, salt, and sugar [11]. Eating mooncakes during the Mid-Autumn Festival has become a Chinese custom. With the rapid development of the food processing industry, a large number of pre-packed mooncakes have become available on the market to meet consumer demand for a wide variety and rich taste. According to T/CNSS001-2018, about 25% of pre-packed mooncakes on sale in China are eligible for the SCL, given that their contents of fat, saturated fatty acids, sugar, and sodium in 100 g do not exceed 25 g, 10 g, 20 g, and 300 mg, respectively [10]. In August 2024, a draft of China’s national guiding standard, Guideline for Iconic Nutrition Labeling for Pre-packed Foods, was released. It includes the SCL as a candidate FOPNL and is currently awaiting public feedback [12]. Thus, it is essential to investigate consumers’ willingness to pay (WTP) for pre-packed mooncakes labeled with the SCL.

There are abundant studies examining consumers’ willingness to pay a premium for foods with nutrition labels. Within these, examples of the specific foods studied include sausages [13,14], breakfast biscuits [15], and cheese [16]; the types of nutrition labels studied include nutrition fact tables [17], nutrition claims [13], and front-of-package nutrition labeling [18,19]. Various methods have been used to reveal consumers’ WTP, including the auction method [20], choice experiments [21,22], and choice-based conjoint experiments [23]. Studies focused on whether consumers were willing to pay a premium, the level of premium that they were willing to pay, the factors influencing WTP, and the heterogeneity, and it was concluded that most consumers are willing to pay a premium for foods with nutrition labeling [19,20,23], although a few are not [21,24,25]. Estimating the response of different consumers’ WTP for labeled foods to changes in premium could provide a reference for manufacturers regarding food pricing and for the government regarding market regulation. However, this has yet to be studied.

The existing literature indicates that consumers’ nutrition knowledge levels represent a direct driver of nutrition label use [26,27,28] and play a moderating role that positively reinforces the impact of health awareness on the effectiveness of nutrition labels [29]. However, the SCL, as an interpretive FOPNL, is suitable for consumers with low nutrition knowledge levels. Hence, it is worth exploring whether consumers’ nutrition knowledge levels enhance or weaken their WTP.

To identify the population heterogeneity in Chinese consumers’ WTP for pre-packed mooncakes labeled with the SCL and the influence of nutrition knowledge levels on their WTP, the semi-double-bounded dichotomous choice contingent value method is adopted in our study to measure Chinese consumers’ WTP. Their response, in terms of WTP, to premiums according to regions, incomes, and BMI classifications is characterized, as well as the moderating effect of nutrition knowledge levels between WTP and premiums. Our findings have potential implications for decision-making in other countries regarding marketing strategies for healthier holiday foods.

## 2. Hypotheses

As demand price theory [30] suggests, the price of foods will rise with elevated premium levels, leading to decreased consumer demand for pre-packed mooncakes. Consumers will derive less utility from the labeled foods and be willing to pay less when the price of mooncakes exceeds their expected value. Thus, Hypothesis 1 is proposed below.

**H1:**  *Consumers’ WTP decreases as the premium for pre-packed mooncakes labeled with the SCL increases*.

The mooncakes in North China are characterized by traditional ingredients, and the main fillings are five fruits, bean paste, or jujube paste, while the mooncakes in South China are characterized by diversified flavors, and the main fillings are lotus seed paste, sesame, ham, or egg yolk [31]. As a whole, the mooncakes in North China are sweeter, greasier, and saltier than those in South China [11]. Moreover, there are regional diet differences in China [32], with consumers from South China generally consuming a lighter diet with lower intake of salt, oil, and sugar than those from North China due to the climate being relatively hot and humid. Compared with consumers from North China, those from South China prefer pre-packed mooncakes low in oil, salt, and sugar [33,34], so the SCL is more likely to draw their attention. It is inferred that the WTP would decrease less for consumers from South China than those from North China as the premium increases. Hypothesis 2 is proposed below.

**H2:**  *The WTP of consumers from South China is less sensitive to the increased premium for pre-packed mooncakes labeled with the SCL than those from North China*.

The demand income theory argues that the consumption of normal commodities increases with individuals’ income [35]. Thus, the consumption of low-oil, -salt, and -sugar pre-packed mooncakes, as a normal commodity, is expected to rise with an increase in individuals’ income. The WTP of consumers will decrease less in those with high income than in those with low income if the premium increases. Hypothesis 3 is proposed below.

**H3:**  *The WTP of high-income consumers is less sensitive to the increased premium for pre-packed mooncakes labeled with the SCL than those with low incomes*.

Obese consumers generally have a higher demand for weight loss and hope to consume less oil, salt, and sugar from foods [36,37], so they are likely to derive a higher expected utility from low-oil, -salt, and -sugar pre-packed mooncakes labeled with the SCL. If the premium increases, obese consumers’ WTP will decrease less than those with healthy weight. Hypothesis 4 is proposed below.

**H4:**  *The WTP of obese consumers is less sensitive to the increased premium for pre-packed mooncakes labeled with the SCL than those with healthy weights*.

Existing studies emphasize that consumers’ increased nutrition knowledge levels contribute to a high usage rate of the nutrition facts table on the back of food packaging, which display information on the unit content of fat, sodium, and sugar, as well as their proportion of daily intake [26,27]. Consumers with higher levels of nutrition knowledge are more likely to assess the healthfulness of pre-packed mooncakes by using the nutrition facts table, which serves as a functional substitute for the SCL. It is speculated that consumers’ nutrition knowledge levels affect their response to the mooncakes’ premium in terms of their WTP. The consumers’ WTP will decrease to a greater extent in response to the increased premium with higher levels of nutrition knowledge. Hypothesis 5 is proposed below.

**H5:**  *The WTP of consumers’ becomes more sensitive to the increased premium for pre-packed mooncakes labeled with the SCL as their level of nutrition knowledge increases*.

## 3. Materials and Methods

### 3.1. Data Collection

The questionnaire was designed based on a literature review and expert consultation and contained 39 questions relating to demographic characteristics, pre-packed mooncake consumption habits, nutrition knowledge levels, and WTP for the pre-packed mooncakes labeled with the SCL (see Supplementary Materials). A presurvey was conducted among 30 randomly selected adults in Beijing on 24 August 2024. Data were collected through a paid online survey on Wenjuanxing (https://www.wjx.cn. accessed on 8 September 2024), a well-known platform in China with a database of 6.2 million registered members.

People in Northeast, North, Northwest, East, Central, Southwest, and South China have distinct eating habits [32]. For this study, one province/autonomous region (referred to as ‘province’ hereafter) was selected from each of the above seven regions, namely Jilin, Inner Mongolia, Shaanxi, Shandong, Henan, Sichuan, and Guangdong, using a stratified random sampling method. Then, the minimum sample size (N = 600) was determined based on an allowable error of 4% and a confidence level of 95%. Finally, 90 adult samples were collected per province to generate 630 valid samples (i.e., 90 samples × 7 provinces) for analysis.

We adopted the quota random sampling method to obtain representative samples. Firstly, the Wenjuanxing member database was stratified according to gender (i.e., male or female), age (18~39 years old, 40~59 years old, or 60 years old and above), education level (primary school or below, junior school, senior school, junior college, or postgraduate and above), annual household disposable income (CNY < 10,000, CNY 10,000~49,999, CNY 50,000~99,999, CNY 100,000~149,999, CNY 150,000~199,999, or CNY ≥ 200,000), and residence (i.e., urban or rural area) of each surveyed province, and the sample number was allocated based on the sample proportion of each layer. Then, the questionnaire link was emailed to randomly selected samples from each layer between 8 and 22 September 2024 (i.e., the peak period for mooncake purchases in China). Prior to data collection, informed written consent was obtained from all participants, who were informed they would receive CNY 8 as a cash incentive for providing accurate and complete responses. Finally, questionnaires continued to be sent until the sample quantity and data quality met the requirements.

**Figure 2:**
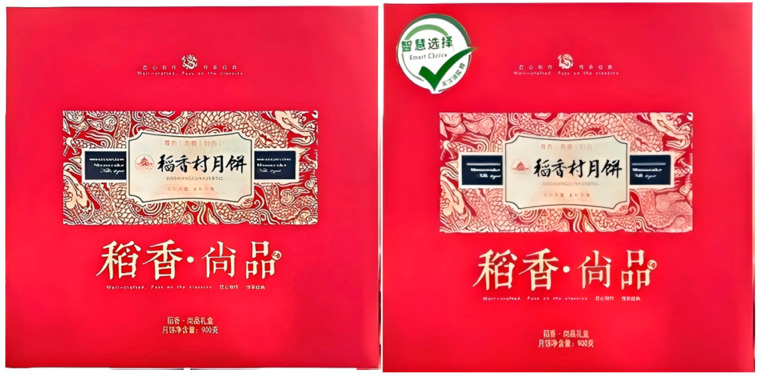
Packaging of pre-packed mooncakes without and with the SCL label. Note: The symbol at the top is the brand logo; the Chinese characters in the middle are Dao xiangcun, a brand of mooncakes; the Chinese characters below the middle are the names of a series of Dao xiangcun products.

The semi-double-bounded dichotomous choice contingent value method was used to investigate respondents’ WTP for pre-packed mooncakes under different premiums. As a method for evaluating the value of public goods’ intangible benefits, this method is used to determine the respondents’ WTP for goods or services in a hypothetical market mainly through a questionnaire survey [38]. Before answering the questions, all respondents were exposed to packages of the same pre-packed mooncakes, either with or without the SCL label (see Figure 2), and informed of the logo’s roles (i.e., it is used to certify low-oil, -salt, and -sugar pre-packed mooncakes). A virtual shopping scene featuring pre-packed mooncakes labeled with the SCL was presented, and the respondents were then asked whether they were willing to pay for the SCL. Questioning on WTP ended when the answer was “no”. The respondents who answered “yes” were then asked to indicate their WTP at 20 different premium levels: >0%, 5%, 10%, 15%, 20%, 25%, 30%, 35%, 40%, 45%, 50%, 55%, 60%, 65%, 70%, 75%, 80%, 85%, 90%, and 95% of the price per unit. The twenty premium levels were displayed in ascending order. When respondents replied “no”, higher premium level(s) were not displayed, and questioning on WTP also ended.

### 3.2. Methods

Consumers’ WTP is expressed as a binary classification (1 = willing to pay; 0 = otherwise). The functional form of the influence of premiums on consumer i’s WTP is expressed as

(1) $${\;\mathrm{WTP}}_{i}=\ln\left(\frac{p}{1-p}\right)=\alpha_{0}+\alpha_{1}{Premium}_{i}+{\alpha_{j}X}_{i}+\varepsilon_{i},$$

where ${\mathrm{WTP}}_{i}$ denotes a latent variable for consumer i’s WTP for pre-packed mooncakes with the SCL under the given premium level (willing to pay if y > 0, and otherwise if y ≤ 0); p indicates consumer i’s willingness probability; ${Premium}_{i}$ is one of the 20 premium levels that consumer i responds to; $\alpha_{1}$ is the influence parameter of premiums on consumer i’s WTP; ${\alpha_{j}X}_{i}$ represents a vector of control variables that influence consumer i’s WTP, including gender, age, education level, etc.; $\alpha_{0}$, $\alpha_{1}$, ⋯, $\alpha_{j}$ are parameters to be estimated, and $\varepsilon_{i}$ is a stochastic disturbance.

The functional form of the influence of premiums on consumer i in group *k*’s WTP is expressed as

(2) $${\;\mathrm{WTP}}_{ki}={\;\beta }_{0}+{\;\beta }_{1}{Premium}_{ki}+{{\;\beta }_{j}X}_{ki}+\epsilon_{ki},$$

where ${\;\mathrm{WTP}}_{ki}$ denotes a latent variable for consumer i in group *k*’s WTP for the mooncakes at the given premium level; *k* indicates a certain region (or income level, a BMI classification); ${Premium}_{ki}$ is one of the 20 premium levels to which consumer i in group *k* responds; $\beta_{1}$ is the influence parameter of premiums on consumer i in group *k* ’s WTP; ${\beta_{j}X}_{ki}$ represents a vector of control variables including gender, age, education level, etc.; $\beta_{0}$, $\beta_{1}$, ⋯, $\beta_{j}$ are parameters to be estimated, and $\epsilon_{ki}$ is a stochastic disturbance.

The functional form of the moderating role of consumer i’s nutrition knowledge level in the influence of premiums on consumer i’s WTP is expressed as

(3) $${\mathrm{WTP}}_{i}={\;\gamma }_{0}+\gamma_{1}{Premium}_{i}+{{\gamma_{2}{Knowledge}_{i}+\gamma_{3}{Premium}_{i}\;\times \;\;{Knowledge}_{i}+\gamma }_{j}X}_{ij}+\theta_{i},$$

where ${\mathrm{WTP}}_{i}$ denotes a latent variable for consumer i’s WTP for pre-packed mooncakes with the SCL under the given premium level; ${Premium}_{i}$ is one of the 20 premium levels to which consumer i responds; ${Knowledge}_{i}$ is consumer i’s nutrition knowledge level. ${Premium}_{i}\times {Knowledge}_{i}$ is the interaction term for the relationship between consumer i’s premium levels and consumer i’s nutrition knowledge level. ${\gamma_{j}X}_{ij}$ represents a vector of control variables including gender, age, education level, etc.; $\gamma_{1}$ + $\gamma_{3}$ is the moderating role parameter of nutrition knowledge levels. $\gamma_{0}$, $\gamma_{1}$, ⋯, $\gamma_{j}$ are parameters to be estimated, and $\theta_{i}$ is a stochastic disturbance.

## 4. Results

### 4.1. Descriptive Statistics

As shown in Table 1, the gender and residence of samples exhibit a similar distribution to that reported in the main data of the 7th National Population Census in 2020 [39]. This validates the representativeness of the overall sample despite the low proportion of the population aged 60 and above and at junior high school level and below.

When respondents indicated their unwillingness to pay a given premium level, then questioning was stopped, and the follow-up survey revealed that the respondents were actually not willing to pay the higher premium. Each of the 630 respondents responded to the 20 given premium levels, resulting in 12,600 (630 samples × 20 responses per person) observations. Table 2 shows that the respondents were 42 years old on average and mostly had a senior school education. For the majority of samples, there were 3~4 people in each family, and the annual household disposable income ranged from CNY 100,000 to 150,000. Respondents’ nutrition knowledge levels were not high, with an average score of only 2.5. Attention was most often paid to the salt, sugar, and fat contents of pre-packed mooncakes and the highly trusted SCL.

**Figure 3:**
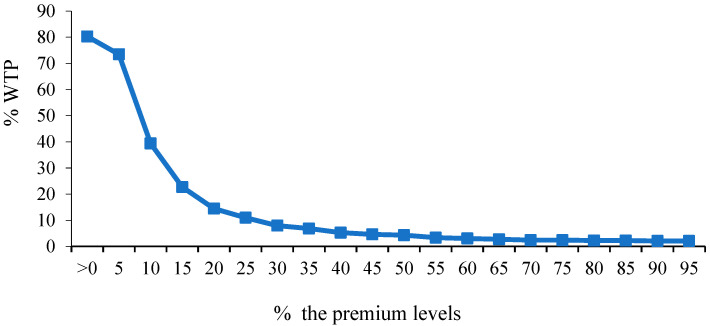
Proportion of consumers’ WTP for pre-packed mooncakes labeled with the SCL under 20 premium levels. Source: The author’s own illustration.

Figure 3 signifies that the proportion of people willing to pay for the SCL decreased with the gradual increase in the premium level. The proportion of people’s WTP for a premium decreased from 80.31% to 2.06% when the premium level increased from >0 to 95%; of particular note, the proportion was less than 50% when the premium level was higher than 10%. This means that more than half of the respondents were willing to pay a premium of no more than 10% of the unit price of pre-packed mooncakes. At present, the average unit price of similar mooncakes (see Figure 2) without the SCL in the retail market is CNY 120. It is inferred that most consumers were only willing to pay a maximum of CNY 12, which is not substantial but could reduce mooncake manufacturers’ unit cost for new product research and development, nutrition testing, packaging redesign, and product marketing to a certain extent.

### 4.2. Empirical Results for the Response of WTP to Premiums

Stata (17.0, StataCorp LLC, College Station, TX, USA) [40] was used for correlation, multicollinearity, and Breusch–Pagan testing of variables before conducting logit regression analysis. There is significant correlation between the independent variable and dependent variables when the variance inflation factor (VIF) of each independent variable was less than 10, which indicates that multicollinearity was not a serious problem. The heteroscedasticity-robust standard error was used in regression analysis due to the existence of heteroscedasticity. The premium levels were not endogenous to respondents’ WTP, since these were exogenously determined as part of the design of the semi-double-bounded dichotomous choice contingent value method.

Table 3 indicates that the coefficient and marginal effect were statistically significant at the 1% significance level, reflecting a higher rate of correct classification. Specifically, the marginal effect of premium levels on respondents’ WTP was −0.007, which suggests that consumers’ WTP decreases (or increases) by 0.7% if the premium level is increased (or decreased) by 1% on average. Additionally, respondents’ age, BMI, family size, concern about the salt, sugar, and fat content of pre-packed mooncakes, and trust in SCL played significantly positive roles, suggesting that consumers who were older, obese, in big families, concerned about the pre-packed mooncakes’ salt, sugar, and fat content, and trusted SCL had a higher willingness to pay.

### 4.3. Empirical Results for the Response of the Subpopulation’s WTP to Premiums

Table 4 indicates that the increasing premium weakened respondents’ WTP for pre-packed mooncakes labeled with the SCL regardless of region, income level, and BMI classification. The increase in premium led to a smaller decrease in the WTP of consumers from South China than those from North China. More specifically, the WTP was least responsive to rising premium levels in Guangdong province (i.e., a marginal effect of −0.005), South China, and more sensitive in Jilin province (−0.007), North China. Overall, the increased premium contributed to a smaller decline in the WTP of consumers with high income than those with low income. For example, the WTP was least responsive to rising premium levels in those with an income above CNY 200,000 (−0.005) but most sensitive in those with an income below CNY 10,000 (−0.007). As for BMI classifications, the WTP was less affected by the increase in premium levels for obese samples (−0.005) than people with healthy weight (−0.007).

### 4.4. The Moderating Effect of Nutrition Knowledge Levels

As shown in Table 5, the regression coefficients of premium levels, nutrition knowledge levels, and their interaction term are statistically significant, suggesting that the increase in the nutrition knowledge levels of individuals significantly enhances the negative influence of premium levels on their WTP. In other words, the negative influence of premium levels on the respondents’ WTP is enhanced with the increase in their nutrition knowledge levels. Specifically, the probability of the premium levels affecting respondents’ WTP decreases by 1.0% (−0.007–0.003) for every 1-point increase in the nutrition knowledge level.

## 5. Discussion

### 5.1. Response of WTP to Premiums

The empirical findings support Hypothesis 1 and highlight that Chinese consumers’ WTP changed to a lesser degree than the premium. This suggests that the demand for pre-packed mooncakes labeled with the SCL is stable since eating mooncakes during the Mid-Autumn Festival is a custom, and there are fewer substitutes in terms of healthier mooncakes. This is in line with the findings of Huang et al. [41], who argued that consumers’ WTP for fresh pork with FOPNL significantly decreases with the increase in premium levels, and the demand is price inelastic. Additionally, the price demand elasticity theory only illustrates that rice and flour are necessities whose demand response to price is inelastic [31], but we revealed that mooncakes are also price inelastic, which enriches the theory. This finding will likely assist manufacturers in understanding the marketing function of the SCL and the market potential of labeled pre-packed mooncakes, motivating them to label pre-packed mooncakes that meet the SCL standard. Therefore, it is essential for mooncake manufacturers to adopt a high-price sales strategy rather than a low-profit and high-turnover strategy, thereby earning higher profits by raising the price of mooncakes labeled with the SCL.

### 5.2. Response of the Subpopulation’s WTP to Premiums

As expected, Hypotheses 2, 3, and 4 are supported, and the impact of premiums on consumers’ WTP varied by region, income, and BMI classification, which is in accordance with a previous study by De-Magistris et al. [16] finding that there is significant population heterogeneity in consumers’ WTP for nutritional claims. In addition, it was confirmed that the WTP of consumers from South China, as well as those with obesity and high income, is not sensitive to increased premiums. This is due not only to their lighter diet [33,34], strong ability to pay [35], and need to lose weight [36,37] but also cultural and social factors. Firstly, mooncakes, as a cultural symbol of happiness, reunion, and good luck in South China, are often given as gifts to relatives and friends [11]. Moreover, mooncakes labeled with the SCL also carry health benefits, so consumers’ WTP was less sensitive to the increased premium. Secondly, due to the effects of the COVID-19 pandemic, consumers with a higher income no longer only aim to grow their wealth but also to pursue good health [42] and may prefer safe, nutritious, and healthy mooncakes. Thirdly, there is currently a prevalent esthetic culture of thinness. Obese people generally have a sense of inferiority and would like to reduce their intake of sugar and fat for weight management, and their WTP was also not sensitive to premiums [43]. The outcomes are expected to be useful for updating programs to promote the SCL in subdivided regions and groups. It is suggested that mooncake manufacturers implement differentiated pricing in different regions and groups. For example, mooncakes with a higher price could be sold to people in South China and people in high-income and weight management groups.

### 5.3. The Moderating Effect of Nutrition Knowledge Levels

Our finding differed from those of studies [26,27] finding that high nutrition knowledge levels are positively associated with high usage of nutrition labeling. We provided evidence for Hypothesis 5 and demonstrated that a consumer’s nutrition knowledge level is a moderating factor that exerts a significantly negative reinforcing effect on the influence of premiums on their WTP. In other words, for every 1-point increase in consumers’ nutrition knowledge level, their WTP decreased by 0.03. An increased nutrition knowledge level enhances the elasticity of consumers’ WTP for the labeled mooncakes. This could be partially due to the fact that while the SCL does actually assist those consumers without a high level of nutrition knowledge in choosing healthy mooncakes, consumers with higher levels of nutrition knowledge and a higher understanding of the nutrition facts table are likely to use the nutrition facts table rather than the SCL to find low-oil, -salt, and -sugar mooncakes without an SCL, thereby avoiding additional expenditure to produce labeled mooncakes. Additionally, factors such as the consumption frequency and the proportion of budget expenditure of foods have been found to influence price demand elasticity in previous studies [30], but we demonstrated that individuals’ nutrition knowledge at the consumer level is another factor. In reality, consumers’ nutrition knowledge levels are improving, which could limit the extent to which the premium on mooncakes would increase, so it is advised that manufacturers set a higher premium for the labeled mooncakes in the short term and gradually reduce this premium over the long term.

### 5.4. Influence of the SCL and Resistance to Its Promotion

The SCL is a practice launched as part of the national oil, salt, and sugar reduction campaign in 2016 with the aim of assisting consumers to easily select healthy pre-packed food. Moreover, the SCL could compensate for the disadvantages resulting from poor comprehension of information in the nutrition facts table, which is the basis for assessing whether to label foods with the SCL. Based on reports of examples encouraging FOPNL, such as the American heart-check mark [44] and the Dutch Choices logo [45], the SCL is expected to reduce Chinese residents’ excessive intake of oil, salt, and sugar from pre-packed foods and lead to a lower incidence of overweight, obesity, and diet-related chronic diseases in the long term.

Overall, consumers were willing to pay premiums for pre-packed mooncakes labeled with the SCL, but this does not mean that all manufacturers may profit as a result. Besides the additional costs of package redesign [46], printing [47], and marketing [48], another possible reason for this is that small manufacturers may attempt to adjust their mooncake recipes according to the SCL standard, which may increase their production costs and raise the price. Due to the fierce competition in the food market, consumers are likely to prefer branded and cost-effective mooncakes to those from small manufacturers, so small manufacturers would be reluctant to supply pre-packed, low-oil, -salt, and -sugar mooncakes. It is inferred that the mandatory promotion of the SCL across China would be hindered by the opposition of small manufacturers.

### 5.5. Limitations

The present work is subject to certain limitations. Firstly, when determining their WTP, respondents were not reminded that the nutrition facts table on the back of the mooncake packaging also provides information on the contents of fat, sodium, and sugar. As a result, respondents, especially those accustomed to using nutrition facts tables to select pre-packed mooncakes, were likely to overestimate the value of the SCL, and their WTP was not affected by increased premiums. Secondly, we failed to examine whether the nutrition knowledge level, as a moderating effect, differed among regions and groups and had further guiding significance for food pricing. Thirdly, education levels are an important driver promoting an individual’s health, but we failed to identify whether respondents’ education levels played a moderating effect on the influence of premiums on their WTP. Therefore, further studies should consider and discuss the role of nutrition knowledge levels in moderating the effect of premiums on WTP in subdivided regions and groups, adopting realistic sales data to reveal respondents’ actual WTP and exploring the moderating effect of education levels.

## 6. Conclusions

We investigated representative samples of Chinese residents to estimate their WTP for pre-packed mooncakes labeled with an SCL and concluded that more than 50% of respondents were willing to pay a premium of up to 10% per unit. There was high demand among respondents for these mooncakes, and their WTP significantly decreased by 0.7% if the premium was increased by 1%. The effect of premiums on WTP varied across provinces, income levels, and BMI classifications. The WTP of respondents who were from South China, obese, or had high incomes was not sensitive to the increase in premium. Nutrition knowledge levels played a moderating role in intensifying the negative influence of premiums, and the rising premium would further reduce WTP with the increase in nutrition knowledge levels. Based on these insights, the following policy recommendations are suggested: It is advised that the SCL be promoted as a national-level FOPNL, but subsidies should be offered to small manufacturers who produce mooncakes with the SCL standard for improving their mooncake recipes. Manufacturers ought to be allowed to set higher prices for labeled mooncakes in South China to target high-income groups, obese populations, and those with a low level of nutrition knowledge. It would be beneficial to enhance consumers’ nutrition knowledge through public education to force manufacturers to improve the nutritional value of mooncakes while using the SCL. Launching encouraging FOPNL, such as the SCL, could be considered in Africa, Southeast Asia, and other places where residents’ overall nutrition knowledge level is low, to help them choose healthier food and thereby contribute to ensuring a healthy diet.

## Figures and Tables

**Table 1:** Sample characteristics.

Characteristic	Item	Samples	Percentage (%)	2020 Population Census Data (%)
Gender	Male	315	50	51.24
	Female	315	50	48.76
Age	18~59 years old	616	97.78	86.30
	60 years old and above	14	2.22	13.70
Education level	Primary school or below	4	0.63	8.10
	Junior school	23	3.65	9.50
	Senior school	428	67.94	55.03
	Junior college	154	24.44	24.61
	Postgraduate and above	21	3.33	2.76
Residence	Urban area	378	60	63.89
	Rural area	252	40	36.11

Source: The author’s own calculation.

**Table 2:** Description of variables and a summary of statistics.

Variable	Definition and Assignment	Mean	Standard Deviation	Min.	Max.	Proportion (%)	Obs.
Dependent							
WTP	No	—	—	—	—	85.37	10,760
	Yes	—	—	—	—	14.63	1840
Independent							
Premium level	%	47.50	28.83	0	95	—	12,600
Gender	Female	—	—	—	—	50.00	315
	Male	—	—	—	—	50.00	315
Age	Years	41.82	11.281	18	81	—	12,600
BMI	Index	23.55	6.417	14.197	64.444	—	12,600
Residence	Rural area	—	—	—	—	40.00	5040
	Urban area	—	—	—	—	60.00	7560
Education level	Primary school or below	—	—	—	—	0.63	80
	Junior school	—	—	—	—	3.65	460
	Senior school	—	—	—	—	67.94	8560
	Junior college or undergraduate	—	—	—	—	24.44	3080
	Postgraduate or above	—	—	—	—	3.33	420
Annual household disposable income (CNY ^a^)	<10,000	—	—	—	—	4.92	620
	10,000~49,999	—	—	—	—	18.10	2280
	50,000~99,999	—	—	—	—	19.52	2460
	100,000~149,999	—	—	—	—	22.70	2860
	150,000~199,999	—	—	—	—	18.41	2320
	≥200,000	—	—	—	—	16.35	2060
Family size	People	3.722	1.210	1	10	—	12,600
Nutrition knowledge level ^b^	Score	2.529	1.161	0	5	—	12,600
Concerned about the salt, sugar, and fat content of pre-packed mooncakes	Not at all	—	—	—	—	0.16	20
	Rarely	—	—	—	—	1.27	160
	Occasionally	—	—	—	—	11.11	1400
	Often	—	—	—	—	44.92	5660
	Always	—	—	—	—	42.54	5360
Trust in SCL	Not at all	—	—	—	—	0	0
	A little	—	—	—	—	4.76	600
	Somewhat	—	—	—	—	26.51	3340
	Mostly	—	—	—	—	51.75	6520
	Very much	—	—	—	—	16.98	2140

Source: The author’s own illustration. Note: ^a^ USD 1 equals CNY 7.064, and EUR 1 equals CNY 7.883 from 8 September to 22 September 2024. ^b^ Each respondent’s nutrition knowledge level was evaluated based on five objective questions, which were designed based on the core content of the Dietary Guidelines for Chinese Residents (2022) [32] (see Supplementary Materials). The respondent received 1 point for each question answered correctly and 0 points for wrong answers. Higher scores indicate a higher nutrition knowledge level.

**Table 3:** Regression results for the influence of premium levels on WTP.

Independent Variables	Coefficient	Marginal Effect
Premium levels	−0.081 *** (0.003)	−0.007 *** (0.001)
Male	−0.153 ** (0.062)	−0.012 ** (0.005)
Age	0.005 * (0.003)	0.001 * (0.001)
BMI	0.023 *** (0.005)	0.002 *** (0.001)
Urban consumers	0.108 (0.069)	0.009 (0.006)
Junior school level	−1.646 *** (0.629)	−0.149 ** (0.065)
Senior school level	−1.309 ** (0.616)	−0.123 * (0.065)
Junior college or undergraduate level	−1.221 ** (0.621)	−0.116 * (0.065)
Postgraduate or above level	−1.529 ** (0.643)	−0.140 ** (0.066)
CNY 10,000~49,999	−0.756 *** (0.185)	−0.066 *** (0.017)
CNY 50,000~99,999	−0.854 *** (0.183)	−0.073 ** (0.017)
CNY 100,000~149,999	−0.432 ** (0.184)	−0.039 ** (0.017)
CNY 150,000~199,999	−0.825 *** (0.183)	−0.071 *** (0.017)
CNY ≥ 200,000	−0.565 *** (0.187)	−0.050 *** (0.017)
Family size	0.082 *** (0.027)	0.007 *** (0.002)
Nutrition knowledge levels	−0.092 *** (0.029)	−0.007 *** (0.002)
Mooncakes’ salt, sugar, and fat content—rarely concerned	0.966 (1.094)	0.021 (0.023)
Mooncakes’ salt, sugar, and fat content—occasionally concerned	2.535 *** (0.896)	0.101 *** (0.015)
Mooncakes’ salt, sugar, and fat content—often concerned	2.793 *** (0.907)	0.120 *** (0.014)
Mooncakes’ salt, sugar, and fat content—always concerned	3.134 *** (0.907)	0.149 *** (0.014)
Some trust in SCL	1.058 *** (0.240)	0.051 *** (0.009)
High trust in SCL	1.879 *** (0.235)	0.113 *** (0.009)
Very high trust in SCL	2.683 *** (0.243)	0.189 *** (0.011)
Constant term	−2.958 *** (0.742)	—
Wald χ^2^	1307.16 ***	
Pseudo R^2^	0.384	
% Correctly classified	90.40	
Obs.	12,600	

Source: The author’s own illustration. Note: Robust standard errors in parentheses; * *p* < 0.1, ** *p* < 0.05, *** *p* < 0.01.

**Table 4:** Results for the response of the subpopulation’s WTP to premiums.

Subsample	Coefficient	Marginal Effect
Regions		
Jilin province (Obs. = 1800)	−0.076 *** (0.007)	−0.007 *** (0.001)
Inner Mongolia province (Obs. = 1800)	−0.120 *** (0.011)	−0.007 *** (0.001)
Shanxi province (Obs. = 1800)	−0.104 *** (0.010)	−0.006 *** (0.001)
Shandong province (Obs. = 1800)	−0.096 *** (0.008)	−0.007 *** (0.001)
Henan province (Obs. = 1800)	−0.135 *** (0.010)	−0.008 *** (0.001)
Sichuan province (Obs. = 1800)	−0.144 *** (0.011)	−0.008 *** (0.001)
Guangdong province (Obs. = 1800)	−0.006 *** (0.005)	−0.005 *** (0.001)
Annual household disposable income (CNY)		
<10,000 (Obs. = 620)	−0.102 *** (0.008)	−0.007 *** (0.001)
10,000~49,999 (Obs. = 2280)	−0.164 *** (0.012)	−0.009 *** (0.001)
50,000~99,999 (Obs. = 2460)	−0.077 *** (0.005)	−0.007 *** (0.001)
100,000~149,999 (Obs. = 2860)	−0.090 *** (0.007)	−0.006 *** (0.001)
150,000~199,999 (Obs. = 2320)	−0.083 *** (0.007)	−0.006 *** (0.001)
≥200,000 (Obs. = 2060)	−0.080 *** (0.010)	−0.005 *** (0.001)
BMI classification		
Thin (Obs. = 920)	−0.112 *** (0.015)	−0.006 *** (0.001)
Normal (Obs. = 8040)	−0.086 *** (0.004)	−0.007 *** (0.001)
Overweight (Obs. = 2160)	−0.161 *** (0.013)	−0.008 *** (0.001)
Obese (Obs. = 1480)	−0.062 *** (0.005)	−0.005 *** (0.001)

Source: The author’s own illustration. Note: Robust standard errors in parentheses; *** *p* < 0.01.

**Table 5:** Results for the moderating effect of nutrition knowledge levels.

Independent Variable	Coefficient	Marginal Effect
Premium levels	−0.057 *** (0.006)	−0.007 *** (0.001)
Nutrition knowledge levels	0.115 ** (0.050)	−0.008 *** (0.002)
Premium levels × Nutrition knowledge levels	−0.011 *** (0.003)	−0.003 *** (0.001)
Control variables	Yes	Yes
Wald χ^2^	1286.03 ***	
Pseudo R^2^	0.388	
% Correctly classified	90.53	
Obs.	12,600	

Source: The author’s own illustration. Note: Robust standard errors in parentheses; ** *p* < 0.05, *** *p* < 0.01.

## Data Availability

The original contributions presented in the study are included in the article/Supplementary Materials, further inquiries can be directed to the corresponding author.

## Supplementary Materials

The following supporting information can be downloaded at: https://www.mdpi.com/article/10.3390/foods13244027/s1, questionnaire.
