# Supplementary material for: Does Nutrition Knowledge Help? Heterogeneity Analysis of Consumers’ Willingness to Pay for Pre-Packed Mooncakes Labeled with the Smart Choice Logo
Source: Foods. 2024 Dec 13;13(24):4027. doi: 10.3390/foods13244027 (PMC11727423; doi:10.3390/foods13244027)
Supplement: Supplementary file 1 [file foods-13-04027-s001.zip › foods-3304632-supplementary.pdf]

The questionnaire

Section 1　Demographic characteristics

Q1-1 Your gender: A. Male　B. Female

Q1-2 Your birth year: ______

Q1-3 Your height: ______cm

Q1-4 Your weight: ______kg

Q1-5 Your residence: A. Urban area　B. Rural area

Q1-6 Your education level: A. Primary school or below B. Junior school C. Senior school D. Junior college or undergraduate　E. Postgraduate or above

Q1-7 Your household's annual disposable income last year : ______Chinese Yuan

A.Less than 10,000 yuan

B.10,000～49,999 yuan

C.50,000～99,999 yuan

D.100,000～149,999 yuan

E.150,000～199,999 yuan

F.200,000 yuan and above

Q1-8 There were: ______ permanent residents in your family last your

Section 2　Pre-packed mooncakes consumption habits

Q2-1 Are you responsible for pre-packed mooncakes shopping at home?

A. always

B. often

C. occasionally

D. rarely

E. not at all

Q2-2 Are you concerned about the salt, sugar and fat content of pre-packed mooncakes?

A. always

B. often

C. occasionally

D. rarely

E. not at all

Q2-3 Do you buy pre-packed mooncakes?

A. always

B. often

C. occasionally

D. rarely

E. not at all

Section 3　Nutrition knowledge level test

Q3-1 What are the negative effects of high energy intake? (Multiple choices)

A. obesity

B. have cardiovascular and cerebrovascular diseases

C. have cancer

D. have diabetes

Q3-2 What is the main function of carbohydrates? (Single choice)

A. main source of energy

B. antioxidant

C. immunity

D. tissue growth

Q3-3 What is the main function of protein? (Single choice)

A. energy offer

B. antioxidants

C. body tissue building and repair

D. appetite improve

Q3-4 What is the maximum daily intake of salt for adults in China? (Single choice)

A. 3 grams

B. 5 grams

C. 8 grams

D. 10 grams

Q3-5 Which nutrients should people with hypertension reduce their intake? (Single choice)

A. protein

B. vitamin

C. fat

D. sodium

Section 4 Willingness to pay for the pre-packed mooncakes labeled with smart choice logos

Figure 1 shows smart choice logos, which certifies prepackaged foods that are low in salt, sugar and fat.

Q4-1 Are you familiar with the logos in Figure 1?

A. very much

B. mostly

C. occasionally

D. rarely

E. not at all

Q4-2 Would you trust the information from the logos in Figure 1?

A. very much

B. mostly

C. occasionally

D. rarely

E. not at all

Q4-3 Do you think the logos in Figure 1 is useful ?

A. very much

B. mostly

C. occasionally

D. rarely

E. not at all

Q4-4 Would you be willing to pay a premium for the pre-packed mooncakes labeled with the logo in Figure 2?

A. Yes

B. No

If your answer is yes, please continue to answer the following questions:

Q4-5 Would you be willing to pay 5% of unit price more for the pre-packed mooncakes labeled with the logo in Figure 2?

A.Yes

B. No

If your answer is yes, please continue to answer the following questions:

Q4-6 Would you be willing to pay 10% of unit price more for the pre-packed mooncakes labeled with the logo in Figure 2?

A.Yes

B. No

If your answer is yes, please continue to answer the following questions:

Q4-7 Would you be willing to pay 15% of unit price more for the pre-packed mooncakes labeled with the logo in Figure 2?

A.Yes

B. No

If your answer is yes, please continue to answer the following questions:

Q4-8 Would you be willing to pay 20% of unit price more for the pre-packed mooncakes labeled with the logo in Figure 2?

A.Yes

B. No

If your answer is yes, please continue to answer the following questions:

Q4-9 Would you be willing to pay 25% of unit price more for the pre-packed mooncakes labeled with the logo in Figure 2?

A.Yes

B. No

If your answer is yes, please continue to answer the following questions:

Q4-10 Would you be willing to pay 30% of unit price more for the pre-packed mooncakes labeled with the logo in Figure 2?

A.Yes

B. No

If your answer is yes, please continue to answer the following questions:

Q4-11 Would you be willing to pay 35% of unit price more for the pre-packed mooncakes labeled with the logo in Figure 2?

A.Yes

B. No

If your answer is yes, please continue to answer the following questions:

Q4-12 Would you be willing to pay 40% of unit price more for the pre-packed mooncakes labeled with the logo in Figure 2?

A.Yes

B. No

If your answer is yes, please continue to answer the following questions:

Q4-13 Would you be willing to pay 45% of unit price more for the pre-packed mooncakes labeled with the logo in Figure 2?

A.Yes

B. No

If your answer is yes, please continue to answer the following questions:

Q4-14 Would you be willing to pay 50% of unit price more for the pre-packed mooncakes labeled with the logo in Figure 2?

A.Yes

B. No

If your answer is yes, please continue to answer the following questions:

Q4-15 Would you be willing to pay 55% of unit price more for the pre-packed mooncakes labeled with the logo in Figure 2?

A.Yes

B. No

If your answer is yes, please continue to answer the following questions:

Q4-16 Would you be willing to pay 60% of unit price more for the pre-packed mooncakes labeled with the logo in Figure 2?

A.Yes

B. No

If your answer is yes, please continue to answer the following questions:

Q4-17 Would you be willing to pay 65% of unit price more for the pre-packed mooncakes labeled with the logo in Figure 2?

A.Yes

B. No

If your answer is yes, please continue to answer the following questions:

Q4-18 Would you be willing to pay 70% of unit price more for the pre-packed mooncakes labeled with the logo in Figure 2?

A.Yes

B. No

If your answer is yes, please continue to answer the following questions:

Q4-19 Would you be willing to pay 75% of unit price more for the pre-packed mooncakes labeled

with the logo in Figure 2?

A.Yes

B. No

If your answer is yes, please continue to answer the following questions:

Q4-20 Would you be willing to pay 80% of unit price more for the pre-packed mooncakes labeled with the logo in Figure 2?

A.Yes

B. No

If your answer is yes, please continue to answer the following questions:

Q4-21 Would you be willing to pay 85% of unit price more for the pre-packed mooncakes labeled with the logo in Figure 2?

A.Yes

B. No

If your answer is yes, please continue to answer the following questions:

Q4-22 Would you be willing to pay 90% of unit price more for the pre-packed mooncakes labeled with the logo in Figure 2?

A.Yes

B. No

If your answer is yes, please continue to answer the following questions:

Q4-23 Would you be willing to pay 95% of unit price more for the pre-packed mooncakes labeled with the logo in Figure 2?

A.Yes

B. No
